# RETRACTED: Starkutė et al. Ascertaining the Influence of Lacto-Fermentation on Changes in Bovine Colostrum Amino and Fatty Acid Profiles. *Animals* 2023 *13*, 3154

**DOI:** 10.3390/ani15070955

**Published:** 2025-03-27

**Authors:** Vytautė Starkutė, Ernestas Mockus, Dovilė Klupšaitė, Eglė Zokaitytė, Saulius Tušas, Ramutė Mišeikienė, Rolandas Stankevičius, João Miguel Rocha, Elena Bartkienė

**Affiliations:** 1 Institute of Animal Rearing Technologies, Lithuanian University of Health Sciences, Tilzes St. 18, LT-47181 Kaunas, Lithuania; vytaute.starkute@lsmu.lt (V.S.); ernestas.mockus@lsmu.lt (E.M.); dovile.klupsaite@lsmu.lt (D.K.); egle.zokaityte@lsmu.lt (E.Z.); saulius.tusas@lsmu.lt (S.T.); ramute.miseikiene@lsmu.lt (R.M.); 2 Department of Food Safety and Quality, Lithuanian University of Health Sciences, Tilzes St. 18, LT-47181 Kaunas, Lithuania; 3 Department of Animal Nutrition, Lithuanian University of Health Sciences, Tilzes St. 18, LT-47181 Kaunas, Lithuania; rolandas.stankevicius@lsmu.lt; 4 Universidade Católica Portuguesa, CBQF—Centro de Biotecnologia e Química Fina—Laboratório Associado, Escola Superior de Biotecnologia, Rua Diogo Botelho 1327, 4169-005 Porto, Portugal; jmfrocha@fc.up.pt; 5 Laboratory for Process Engineering, Environment, Biotechnology and Energy (LEPABE), Faculty of Engineering, University of Porto (FEUP), Rua Dr. Roberto Frias, s/n, 4200-465 Porto, Portugal; 6 Associate Laboratory in Chemical Engineering (ALiCE), Faculty of Engineering, University of Porto (FEUP), Rua Dr. Roberto Frias, s/n, 4200-465 Porto, Portugal

The Journal *Animals* retracts the article titled “Ascertaining the Influence of Lacto-Fermentation on Changes in Bovine Colostrum Amino and Fatty Acid Profiles” [1], cited above.

Following publication, concerns were brought to the attention of the Editorial Office regarding overlap between this publication [1] and an earlier article [2] published by a similar authorship group.

Adhering to our standard procedure, an investigation was conducted by the Editorial Office and Editorial Board, which included an evaluation of the scientific content and findings of the paper. While the authors fully cooperated with the investigation through the clarification of the complaint, the Editorial Board confirmed a high level of redundancy between these publications [1,2], without appropriate acknowledgement or citation. Furthermore, the Editorial Board identified concerns relating to the completeness of the analysis and the degree to which the study addresses the stated objectives of this study.

Considering the redundancy and the concerns relating to the analysis and conclusions presented in this publication [1], the Editorial Board have determined that a correction would not appropriately address these issues. Consequently, the Editorial Board has decided to retract this article as per MDPI’s retraction policy (https://www.mdpi.com/ethics#_bookmark30, accessed on 19 March 2025).

This retraction was approved by the Editor-in-Chief of the journal *Animals*.

The authors did not agree to this retraction.
